# Supplementary material for: Integrin-associated transcriptional characteristics of circulating tumor cells in breast cancer patients
Source: PeerJ. 2024 Jan 18;12:e16678. doi: 10.7717/peerj.16678 (PMC10800097; doi:10.7717/peerj.16678)
Supplement: Supplemental Information 1 [file peerj-12-16678-s001.pdf]

Supplementary Table 1. Clinicopathological characteristics of breast cancer patients

Cases	Age at diagnosis (yr)	Grade	TNM	Focality	Molecular subtype	ER	PR	HER2	Ki-67 (%)	Neoadjuvant chemotherapy
1	37	3	T2N2M0	UF	TN	-	+	0	80	Yes
2	50	3	T2N0M0	MF	Lum B HER2-	+	+	1+	38	No
3	53	2	T1N0M0	MF	TN	-	-	0	87	No
4	64	2	T2N1M0	UF	Lum B HER2-	+	+	1+	30	No
5	64	2	T2N0M0	UF	Lum B HER2+	+	+	3+	21	Yes
6	56	1	T4N3M0	UF	Lum B HER2-	+	-	1+	42	No
7	44	2	T2N1M0	UF	Lum B HER2-	+	+	0	20	No
8	58	3	T4N1M0	UF	TN	-	-	2+ (FISH-)	52	Yes
9	48	3	T2N2M0	UF	TN	-	-	0	36	Yes
10	58	2	T2N1M0	UF	Lum B HER2-	+	+	1+	43	No
11	71	3	T2N1M0	UF	Lum B HER2-	+	0	0	85	No
12	76	1	T2NxM0	UF	Lum A HER2-	+	+	1+	7	No
13	45	2	T2N0M0	UF	Lum A HER2-	+	+	1+	14	No
14	60	2	T3N1M0	UF	Lum B HER2-	+	+	0	32	ND
15	62	1	T2N1M0	UF	Lum B HER2-	+	+	0	44	Yes
16	36	2	T2NxM0	MF	Lum B HER2-	+	+	0	35	Yes
17	45	2	T2N0M0	UF	TN	-	-	0	77	Yes
18	68	3	T2NxMx	UF	Lum B HER2-	+	0	-	33	Yes
19	60	1	T2N0M0	UF	Lum B HER2-	+	+	2+ (FISH-)	38	No
20	55	2	T4N1M0	UF	HER2+	-	-	3+	18	Yes

UF, unifocal; MF, multifocal; Lum, luminal; TN, triple-negative; yr, years; ER, estrogen receptors; PR, progesterone receptors; TNM, tumor-node-metastasis classification; "+", presence; "-", absence; FISH, fluorescence in situ hybridization

Supplementary Table 2. Expression of stemness/EMT and proliferation genes in CTCs of breast cancer patients

	CD133/ PROM1	*ALDH1A1*	*CD44/ CD24*	*SNAI1*	*SNAI2*	*KLF4*	*OCT4/ POU5F1*	*MYC*	*CDH1*	*CDH2*	*VIM*	*ZEB1*	*ZEB2*	*MKI67*
1. CTCs expressing isolated integrin subunits, n=143	1/143 (0.7%)	0/143 (0.0%)	13/143 (9.1%)	0/143 (0.0%)	0/143 (0.0%)	1/143 (0.7%)	0/143 (0.0%)	0/143 (0.0%)	16/143 (11.2%)	0/143 (0.0%)	56/143 (39.2%)	3/143 (2.1%)	39/143 (27.3%)	0/143 (0.0%)
2. CTCs expressing isolated integrin α-subunits, n=81	1/81 (1.2%)	2/81 (2.5%)	16/81 (19.8%)	1/81 (1.2%)	0/81 (0.0%)	2/81 (2.5%)	0/81 (0.0%)	0/81 (0.0%)	7/81 (8.6%)	0/81 (0.0%)	38/81 (46.9%)	2/81 (2.5%)	30/81 (37.0%) **p=0.008**	1/81 (1.2%)
3. CTCs expressing different heterodimers containing β1-subunit without other β-subunits, n=8	0/8 (0.0%)	1/8 (12.5%) **p=0.043***	1/8 (12.5%)	0/8 (0.0%)	0/8 (0.0%)	1/8 (12.5%)	1/8 (12.5%) **p=0.043**	0/8 (0.0%)	1/8 (12.5%)	0/8 (0.0%)	3/8 (37.5%)	0/8 (0.0%)	3/8 (37.5%)	1/8 (12.5%)
4. CTCs expressing different heterodimers containing β2-subunit without other β-subunits, n=2	0/2 (0.0%)	0/2 (0.0%)	1/2 (50.0%)	0/2 (0.0%)	0/2 (0.0%)	0/2 (0.0%)	0/2 (0.0%)	0/2 (0.0%)	1/2 (50.0%)	0/2 (0.0%)	2/2 (100.0%)	0/2 (0.0%)	2/2 (100.0%) **p=0.042**	0/2 (0.0%)
5. CTCs expressing different heterodimers containing β3-subunit without other β-subunits, n=11	0/11 (0.0%)	0/11 (0.0%)	0/11 (0.0%)	0/11 (0.0%)	0/11 (0.0%)	0/11 (0.0%)	0/11 (0.0%)	0/11 (0.0%)	0/11 (0.0%)	0/11 (0.0%)	1/11 (9.1%)	0/11 (0.0%)	1/11 (9.1%)	0/11 (0.0%)
6. CTCs expressing different β-subunits with at least one potential heterodimer, n=38	0/38 (0.0%)	0/38 (0.0%)	4/38 (10.5%)	0/38 (0.0%)	0/38 (0.0%)	0/38 (0.0%)	0/38 (0.0%)	0/38 (0.0%)	1/38 (2.6%)	0/38 (0.0%)	9/38 (23.7%)	0/38 (0.0%)	14/38 (36.8%) **p=0.034**	0/38 (0.0%)
7. CTCs expressing β4-subunit along with other subunits or heterodimer, n=3	0/3 (0.0%)	1/3 (33.3%) **p=0.017**	0/3 (0.0%)	0/3 (0.0%)	0/3 (0.0%)	0/3 (0.0%)	0/3 (0.0%)	1/3 (33.3%) **p=0.038**	1/3 (33.3%)	0/3 (0.0%)	1/3 (33.3%)	1/3 (33.3%)	2/3 (66.6%)	0/3 (0.0%)
8. CTCs expressing genes of full-fledged integrin α6β4, n=5	1/5 (20%) **p=0.027**	1/5 (20%) **p=0.027**	1/5 (20%)	0/5 (0%)	0/5 (0.0%)	3/5 (60%) **p=0.033**	0/5 (0.0%)	2/5 (40%) **p=0.002**	0/5 (0.0%)	1/5 (20%) **p=0.027**	5/5 (100%) **p=0.01**	4/5 (80%) **p<0.001**	2/5 (40%)	1/5 (10.0%) **p=0.06**
9. CTCs without integrins subunits gene expression, n=154	0/154 (0.0%)	0/154 (0.0%)	20/154 (13.0%)	1/154 (0.65%)	0/154 (0.0%)	2/154 (1.3%)	0/154 (0.0%)	1/154 (0.65%)	20/154 (13.0%)	0/154 (0.0%)	61/154 (39.6%)	6/154 (3.9%)	31/154 (20.1%)	1/154 (0.65%)

*Note: The table shows significant differences compared to the CTCs without integrins gene expression (group 9).

Supplementary Table 3. Gene expression of laminin subunits in CTCs of breast cancer patients

	LAMA4	*LAMA5*	*LAMB1*	*LAMB2*	*LAMB3*	*LAMC1*
1. CTCs expressing isolated integrin subunits, n=143	0/143 (0.0%)	1/143 (0.0%)	1/143 (0.0%)	0/143 (0.0%)	1/143 (0.0%)	0/143 (0.0%)
2. CTCs expressing isolated integrin α-subunits, n=81	0/81 (0.0%)	1/81 (1.2%)	2/81 (2.4%)	0/81 (0.0%)	0/81 (0.0%)	2/81 (2.4%)
3. CTCs expressing different heterodimers containing β1-subunit without other β-subunits, n=8	0/8 (0.0%)	1/8 (0.0%)	1/8 (0.0%)	0/8 (0.0%)	0/8 (0.0%)	1/8 (12.5%)
4. CTCs expressing different heterodimers containing β2-subunit without other β-subunits, n=2	0/2 (0.0%)	0/2 (0.0%)	0/2 (0.0%)	0/2 (0.0%)	0/2 (0.0%)	0/2 (0.0%)
5. CTCs expressing different heterodimers containing β3-subunit without other β-subunits, n=11	0/11 (0.0%)	0/11 (0.0%)	0/11 (0.0%)	0/11 (0.0%)	0/11 (0.0%)	0/11 (0.0%)
6. CTCs expressing different β-subunits with at least one potential heterodimer, n=38	0/38 (0.0%)	0/38 (0.0%)	1/38 (0.0%)	0/38 (0.0%)	0/38 (0.0%)	0/38 (0.0%)
7. CTCs expressing β4-subunit along with other subunits or heterodimer, n=3	0/3 (0.0%)	0/3 (0.0%)	0/3 (0.0%)	0/3 (0.0%)	0/3 (0.0%)	0/3 (0.0%)
8. CTCs expressing genes of full-fledged integrin $\alpha6\beta4$, n=5	5/5 (100.0%) **p<0.001***	1/5 (20.0%) **p=0.028**	5/5 (100.0%) **p<0.001**	3/5 (60.0%) **p<0.001**	1/5 (20.0%) **p=0.028**	4/5 (80.0%) **p<0.001**
9. CTCs without integrins subunits gene expression, n=154	0/154 (0.0%)	0/154 (0.0%)	0/154 (0.0%)	0/154 (0.0%)	0/154 (0.0%)	1/154 (0.6%)

*Note: The table shows significant differences compared to the CTCs without integrins gene expression (group 9).

Supplementary Table 4. Gene expression of chemokines/their receptors and inflammatory factors in CTCs of breast cancer patients

	TGFB1	*VEGFA*	*CCL2*	*CXCR7/ ACKR3*	*IL1B*	*CXCL8/ IL8*	*IL18*	*IL6*	*CASP1*	*NLRP3*	*ASC/ PYCARD*
1. CTCs expressing isolated integrin subunits, n=143	72/143 (50.3%) **p<0.001***	1/143 (0.7%)	0/143 (0.0%)	0/143 (0.0%)	5/143 (3.5%)	3/143 (2.1%)	7/143 (4.9%) **p=0.005**	0/143 (0.0%)	2/143 (1.4%)	1/143 (0.7%)	21/143 (14.7%)
2. CTCs expressing isolated integrin α-subunits, n=81	28/81 (34.6%) **p=0.015**	0/81 (0.0%)	0/81 (0.0%)	0/81 (0.0%)	3/81 (3.7%)	2/81 (2.5%)	5/81 (6.2%)	0/81 (0.0%)	1/81 (1.2%)	2/81 (2.4%)	11/81 (13.6%)
3. CTCs expressing different heterodimers containing β1-subunit without other β-subunits, n=8	3/8 (37.5%)	0/8 (0.0%)	0/8 (0.0%)	0/8 (0.0%)	0/8 (0.0%)	0/8 (0.0%)	0/8 (0.0%)	0/8 (0.0%)	0/8 (0.0%)	0/8 (0.0%)	1/8 (12.5%)
4. CTCs expressing different heterodimers containing β2-subunit without other β-subunits, n=2	0/2 (0.0%)	1/2 (0.0%)	0/2 (0.0%)	0/2 (0.0%)	1/2 (50.0%) **p=0.022**	1/2 (50.0%) **p=0.022**	0/2 (0.0%)	1/2 (50.0%) **p=0.022**	0/2 (0.0%)	1/2 (50.0%) **p=0.022**	0/2 (0.0%)
5. CTCs expressing different heterodimers containing β3-subunit without other β-subunits, n=11	8/11 (72.7%) **p=0.006**	0/11 (0.0%)	0/11 (0.0%)	0/11 (0.0%)	0/11 (0.0%)	0/11 (0.0%)	0/11 (0.0%)	0/11 (0.0%)	0/11 (0.0%)	0/11 (0.0%)	0/11 (0.0%)
6. CTCs expressing different β-subunits with at least one potential heterodimer, n=38	35/38 (92.1%) **p<0.001**	1/38 (2.6%)	0/38 (0.0%)	0/38 (0.0%)	1/38 (2.6%)	2/38 (5.2%) **p<0.001**	1/38 (2.6%)	0/38 (0.0%)	0/38 (0.0%)	0/38 (0.0%)	2/38 (5.2%)
7. CTCs expressing β4-subunit along with other subunits or heterodimer, n=3	1/3 (33.3%)	1/3 (33.3%) **p=0.038**	0/3 (0.0%)	0/3 (0.0%)	0/3 (0.0%)	0/3 (0.0%)	0/3 (0.0%)	0/3 (0.0%)	0/3 (0.0%)	0/3 (0.0%)	1/3 (33.3%)
8. CTCs expressing genes of full-fledged integrin α6β4, n=5	5/5 (100.0%) **p=0.003**	3/5 (60.0%) **p<0.001**	2/5 (40.0%) **p<0.001**	5/5 (100.0%) **p<0.001**	0/5 (0.0%)	1/5 (20.0%) **p=0.054**	0/5 (0.0%)	1/5 (20.0%) **p=0.028**	1/5 (20.0%) **p= 0.028**	0/5 (0.0%)	1/5 (20.0%)
9. CTCs without integrins subunits gene expression, n=154	46/154 (29.9%)	1/154 (0.6%)	0/154 (0.0%)	0/154 (0.0%)	1/154 (0.6%)	1/154 (0.6%)	0/154 (0.0%)	0/154 (0.0%)	0/154 (0.0%)	0/154 (0.0%)	16/154 (9.0%)

*Note: The table shows significant differences compared to the CTCs without integrins gene expression (group 9).

Supplementary Table 5. Gene expression of invasion markers in CTCs of breast cancer patients

	RAC1	***RHOC***	***ROCK1***	***MMP1***	***MMP2***
1. CTCs expressing isolated integrin subunits, n=143	53/143 (37.0%)	15/143 (10.5%)	20/143 (14.0%)	0/143 (0.0%)	0/143 (0.0%)
2. CTCs expressing isolated integrin α-subunits, n=81	25/81 (30.9%)	7/81 (8.6%)	17/81 (21.0%) **p=0.020**	0/81 (0.0%)	0/81 (0.0%)
3. CTCs expressing different heterodimers containing β1-subunit without other β-subunits, n=8	2/8 (25.0%)	1/8 (12.5%)	1/8 (12.5%)	0/8 (0.0%)	0/8 (0.0%)
4. CTCs expressing different heterodimers containing β2-subunit without other β-subunits, n=2	1/2 (50.0%)	0/2 (0.0%)	1/2 (50.0%)	0/2 (0.0%)	0/2 (0.0%)
5. CTCs expressing different heterodimers containing β3-subunit without other β-subunits, n=11	6/11 (54.5%)	0/11 (0.0%)	0/11 (0.0%)	0/11 (0.0%)	0/11 (0.0%)
6. CTCs expressing different β-subunits with at least one potential heterodimer, n=38	27/38 (71.1%) **p<0.001***	12/38 (31.6%) **p=0,001**	10/38 (26.3%) **p=0.013**	0/38 (0.0%)	0/38 (0.0%)
7. CTCs expressing β4-subunit along with other subunits or heterodimer, n=3	1/3 (33.3%)	0/3 (0.0%)	2/3 (66.7%) **p=0.027**	0/3 (0.0%)	0/3 (0.0%)
8. CTCs expressing genes of full-fledged integrin $\alpha6\beta4$, n=5	5/5 (100.0%) **p=0.002**	5/5 (100.0%) **p<0.001**	4/5 (80.0%) **p=0.001**	1/5 (20.0%) **p=0.028**	5/5 (100.0%) **p<0.001**
9. CTCs without integrins subunits gene expression, n=154	45/154 (29.2%)	15/154 (9.7%)	15/154 (9.7%)	0/154 (0.0%)	0/154 (0.0%)

*Note: The table shows significant differences compared to the CTCs without integrins gene expression (group 9).

Supplementary Table 6. Gene expression of epithelial markers in CTCs of breast cancer patients

	EPCAM	*KRT10*	*KRT5*	*KRT8*	*KRT7*	*KRT18*	*KRT19*	*MUC1*	*CDH1*	*ERBB2*
1. CTCs expressing isolated integrin subunits, n=143	1/143 (0.7%)	15/143 (10.5%)	42/143 (29.4%)	55/143 (38.5%)	0/143 (0.0%)	30/143 (21.0)	1/143 (0.7%)	2/143 (1.4%)	16/143 (11.2%)	0/166 (0.0%)
2. CTCs expressing isolated integrin α-subunits, n=81	5/81 (6.2%)	17/81 (21.0%)	37/81 (45.7%)	19/81 (23.4%)	1/81 (1.2%)	14/81 (17.3%)	0/81 (0.0%)	3/81 (3.7%)	7/81 (8.6%)	0/81 (0.0%)
3. CTCs expressing different heterodimers containing β1-subunit without other β-subunits, n=8	1/8 (12.5%)	1/8 (12.5%)	3/8 (37.5%)	2/8 (25.0%)	0/8 (0.0%)	2/8 (25.0%)	0/8 (0.0%)	1/8 (12.5%)	1/8 (12.5%)	1/8 (12.5%) **p=0.049**
4. CTCs expressing different heterodimers containing β2-subunit without other β-subunits, n=2	0/2 (0.0%)	0//2 (0.0%)	0/2 (0.0%)	0/2 (0.0%)	1/2 (50.0%)	0/2 (0.0%)	0/2 (0.0%)	0/2 (0.0%)	1/2 (50.0%)	0/2 (0.0%)
5. CTCs expressing different heterodimers containing β3-subunit without other β-subunits, n=11	0/11 (0.0%)	0/11 (0.0%)	0/11 (0.0%)	7/11 (63.6%) **p=0.005**	0/11 (0.0%)	4/11 (36.4%)	0/11 (0.0%)	0/11 (0.0%)	0/11 (0.0%)	0/11 (0.0%)
6. CTCs expressing different β-subunits with at least one potential heterodimer, n=38	0/38 (0.0%)	2/38 (5.3%)	5/38 (5.3%) **p=0.001**	18/38 (47.4%) **p=0.004**	0/38 (0.0%)	15/38 (39.5%) **p=0.017**	0/38 (0.0%)	0/38 (0.0%)	1/38 (2.6%)	0/38 (0.0%)
7. CTCs expressing β4-subunit along with other subunits or heterodimer, n=3	1/3 (33.3%)	1/3 (33.3%)	1/3 (33.3%)	0/3 (0.0%)	0/3 (0.0%)	0/3 (0.0%)	0/3 (0.0%)	0/3 (0.0%)	1/3 (33.3%)	0/3 (0.0%)
8. CTCs expressing genes of full-fledged integrin α6β4, n=5	0/5 (0.6%)	3/5 (60.0%) **p=0.013***	0/5 (0.0%)	3/5 (60.0%)	0/5 (0.0%)	5/5 (100.0%) **p<0.001**	2/5 (40.0%) **p=0.011**	0/5 (0.0%)	0/5 (0.0%)	0/5 (0.0%)
9. CTCs without integrins subunits gene expression, n=154	4/154 (2.6%)	16/154 (10.4%)	70/154 (45.5%)	34/154 (22.1%)	1/154 (0.6%)	30/154 (19.5%)	4/154 (2.6%)	2/154 (1.2%)	20/154 (13.0%)	0/154 (0.0%)

*Note: The table shows significant differences compared to the CTCs without integrins gene expression (group 9).
